# No Differential Regulation of Dopamine Transporter (DAT) and Vesicular Monoamine Transporter 2 (VMAT2) Binding in a Primate Model of Parkinson Disease

**DOI:** 10.1371/journal.pone.0031439

**Published:** 2012-02-16

**Authors:** LinLin Tian, Morvarid Karimi, Susan K. Loftin, Chris A. Brown, HuChuan Xia, JinBin Xu, Robert H. Mach, Joel S. Perlmutter

**Affiliations:** 1 Department of Neurology, Washington University, St. Louis, Missouri, United States of America; 2 Department of Radiology, Washington University, St. Louis, Missouri, United States of America; 3 Department of Neurobiology, Washington University, St. Louis, Missouri, United States of America; 4 Department of Occupational Therapy, Washington University, St. Louis, Missouri, United States of America; 5 Department of Physical Therapy, Washington University, St. Louis, Missouri, United States of America; University of São Paulo, Brazil

## Abstract

Radioligands for DAT and VMAT2 are widely used presynaptic markers for assessing dopamine (DA) nerve terminals in Parkinson disease (PD). Previous *in vivo* imaging and postmortem studies suggest that these transporter sites may be regulated as the numbers of nigrostriatal neurons change in pathologic conditions. To investigate this issue, we used *in vitro* quantitative autoradioradiography to measure striatal DAT and VMAT2 specific binding in postmortem brain from 14 monkeys after unilateral internal carotid artery infusion of 1-Methyl-4-Phenyl-1,2,3,6-tetrahydropyridine (MPTP) with doses varying from 0 to 0.31 mg/kg. Quantitative estimates of the number of tyrosine hydroxylase (TH)-immunoreactive (ir) neurons in substantia nigra (SN) were determined with unbiased stereology, and quantitative autoradiography was used to measure DAT and VMAT2 striatal specific binding. Striatal VMAT2 and DAT binding correlated with striatal DA (*r_s_* = 0.83, *r_s_* = 0.80, respectively, both with *n* = 14, *p*<0.001) but only with nigra TH-ir cells when nigral cell loss was 50% or less (*r* = 0.93, *n* = 8, *p* = 0.001 and *r* = 0.91, *n* = 8, *p* = 0.002 respectively). Reduction of VMAT2 and DAT striatal specific binding sites strongly correlated with each other (*r* = 0.93, *n* = 14, *p*<0.0005). These similar changes in DAT and VMAT2 binding sites in the striatal terminal fields of the surviving nigrostriatal neurons demonstrate that there is no differential regulation of these two sites at 2 months after MPTP infusion.

## Introduction

Selective degeneration of dopaminergic neurons in the substantia nigra pars compacta (SNpc) combined with striatal DA deficiency produces the major motor manifestations of idiopathic PD [1], [2]. Reduction of dopaminergic neurons in PD patients and animal models of parkinsonism leads to substantial loss of the pre-synaptic markers DAT, TH and VMAT2. However, there are discrepancies in the magnitude of the changes in these presynaptic markers, and differential regulation of these sites may account for these discrepancies [3]–[5].

Previous studies have used *in vitro* or *in vivo* methods to measure the effects of nigrostriatal deficiency on loss of striatal DAT and VMAT2. One *in vitro* autoradiographic study from PD patients revealed greater striatal loss of DAT than VMAT2 specific binding sites [3]. However, studies with MPTP-treated rodents have produced conflicting data on the extent of reduction of striatal DAT and VMAT2. Some studies reported greater loss of striatal DAT compared to VMAT2 [4], whereas others reported no significant difference in loss of these specific binding sites [6]. *In vivo* radiotracer imaging studies of VMAT2 and DAT have revealed conflicting results as well. Some report greater loss of striatal DAT [5] while others found greater loss of VMAT2 in PD patients [7]. In addition, there are discrepancies in nonhuman primate models of PD [8] with variable changes in DAT and VMAT2 loss [8]–[10].

A number of factors complicate interpretation of data from human studies including studying subjects at different clinical stages of disease, disparities in length of drug treatment and differences in radiotracer specificity. Animal models of PD have been used to overcome many of these problems; however, differences in experimental methodology including limited range of severity of neurotoxin-induced neuronal loss, varying specificity of radioligands and insufficient time after neurotoxin delivery to achieve a stable course may account for varying results.

The purpose of the present study, therefore, was to examine the changes of DAT and VMAT2 binding sites in adult male monkeys who had no other exposure to experimental drugs after unilateral internal carotid artery infusion of MPTP with doses varying from 0 to 0.31 mg/kg. This infusion schedule produced a wide range of severity of unilateral stable hemi-parkinsonism while minimizing exposure of the contralateral hemisphere to MPTP [11]–[13]. [^3^H]WIN 35,428 was chosen to label DAT [14] and (+)-[^3^H] dihydrotetrabenazine (DTBZ) was used for VMAT2 [15], [16]. This experimental design permitted comparison of changes in DAT and VMAT2 binding sites in a full spectrum of nigrostriatal neuronal loss induced by MPTP. Finally, we also determined the relationship of DAT and VMAT2 binding to stereologic counts of nigral neurons and striatal DA.

## Methods

### Ethics Statement

We used the minimum number of animals necessary for this research and followed all relevant national and international guidelines. In accordance with the recommendations of the Weatherall report “The use of non-human primates in research,” we took all steps to ameliorate suffering in our work with non-human primates. The welfare of the animals conformed to the requirements of National Institutes of Health (NIH), and we followed the guidelines prescribed by the NIH Guide to Laboratory Animal Care. This work was conducted at the Nonhuman Primate Facility of Washington University in St. Louis with approval from its Institutional Animal Care and Use Committee (IACUC). The protocol number is 20110161, last approved on 8 July 2011. All animals were housed in the same room in cages meeting or exceeding the stipulated size requirements. Animals were maintained in facilities with 12-hour dark and light cycles, given access to foold and water ad libitum; all animals were equally engaged with a variety of psychologically enriching tasks such as watching movies or playing with appropriate toys. No animal was knowingly exposed to potential infection. Humane endpoints were pre-defined in this protocol and applied as a measure if necessary to reduce any discomfort.

### Subjects

Fourteen male macaques (*Macaca fascicularis* and *Macaca nemestrina*), between the ages of 3.5 and 6.5 years old (mean = 5.4±1.0), served as subjects.

### MPTP infusion

MPTP (Sigma, St. Louis, MO) was administered as described by Tabbal et al., [13]. A single MPTP dose of 0 (normal saline with no MPTP) to 0.31 mg/kg was infused into the right internal carotid artery. Location was confirmed through angiograms before and after MPTP infusion [17]. After the procedure, animals were continuously observed until able to care for themselves. Animals received no dopaminergic drugs at any time.MPTP and potentially toxic metabolites were decontaminated and discarded as follows. At the end of the MPTP infusion, all work surfaces and equipment were decontaminated immediately with a 2.5% solution of hypochlorite bleach; any unwanted remaining solution of MPTP, plastic products (syringes, tubing, etc.), and dry waste were treated with bleach, and disposed as hazardous chemical waste through Washington University in St. Louis Environment, Health and Safety (EHS) chemical waste disposal program. After administration of MPTP, trained staff lined the base of the cage and the drop pan with plastic-backed absorbent pads; sprayed these pads and drop pans with bleach, allowed them to soak for 10 min, then disposed this biohazardous waste daily for 5 days post MPTP.

### Tissue Processing

The monkeys were euthanized two months after MPTP infusion with a lethal overdose of pentobarbital (100 mg/kg, i.v.) (Butler Schein Animal Health, Dublin, OH). The brains were removed within 10 minutes, and the hemispheres and midbrain were separated. Standard punch biopsies were taken from caudate and putamen, quickly frozen on dry ice snow and saved at −80°C for DA and DA metabolite quantification. A coronal slab from each hemisphere was rapidly frozen in liquid nitrogen vapor and then stored at −80°C for autoradiography. Coronal sections, 20 µm thick, were cut on a cryostat (Microm HM 550 series, Thermo Fisher Scientific Inc, Waltham, MA), mounted on superfrost plus glass slides (Thermo Fisher Scientific, Waltham, MA), and stored at −80°C until used in quantitative autoradiography. The entire midbrain was post-fixed for 7 days in 4% paraformaldehyde in 0.1 M phosphate-buffered saline (PBS) before transfer to 30% sucrose for 7 days, and then cut on a freezing microtome into serial, free-floating 50 µm-thick transverse sections. A random series of every 6^th^ section was processed for TH immunocytochemistry.

### TH Immunohistochemistry

We used TH-immunostaining to identify dopaminergic neurons. Sections were treated for 20 minutes in 0.3% hydrogen peroxide (Fisher Scientific, Fair Lawn, NJ), washed 3 times in PBS, and blocked in PBS with 2% normal goat serum and 0.3% Triton X-100 for 2 hours prior to overnight incubation at 4°C with rabbit anti-TH (1∶1000; Chemicon International, Inc., Temecula, CA) . After washes with PBS (5 min, 3 times), the sections were reacted at room temperature for 60 minutes with biotin-conjugated universal secondary antibody provided in Universal Vectastain (1∶200; Vector Laboratories, Burlingame, CA). After subsequent washes in PBS, the sections were incubated in streptavidin-biotin complex (Vector Laboratories, Burlingame, CA) for 60 min at room temperature. Following thorough rinsing with PBS, staining was visualized by incubation in 3, 3′-diaminobenzidine solution with nickel enhancement (Vector Laboratories, Burlingame, CA). After immunostaining, floating tissue sections were mounted on gelatin-coated glass slides and counterstained with cresyl violet. Sections without primary antibody served as negative controls.

### Unbiased stereologic counting of nigrostriatal neurons

**Figure 1:**
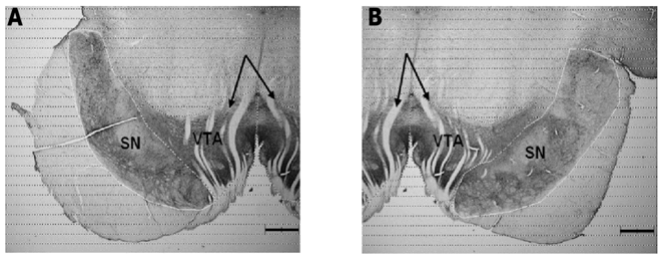
SN outlined on a TH-immunostained transverse slice with 2× magnification. Typical pictures were taken on the left (A) and right (B) side of the same slice separately with SN and ventral tegmental area (VTA) outlined. SN lies ventral and lateral to VTA. The 3^rd^ cranial nerve fibers are indicated by arrows. Scale bar: 1 cm.

We applied unbiased stereology to the immunostained sections to quantify the number of dopaminergic neurons. Procedures followed those defined by Gunderson, et al., [18], [19] using an Olympus BX41 Microscope with a proscan stage kit and DP70 digital camera. The SN was defined as the region ventral to the medial leminiscus, dorsal to the cerebral peduncle and lateral to the third cranial nerve (Figure 1). The computer-assisted stereology (CAST, version 3.2.10.0; Visiopharm, Hoersholm, Denmark) was utilized to achieve a random sampling of 5.22% (frame length/step length) of the total region of interest area with an 80×80 µm^2^ counting frame and height of 22 µm with a 3 µm top guard zone. TH-ir cells were identified; total tissue thickness ranged from 25 to 30 µm due to shrinkage during processing. Weighted thickness averaging was applied to the calculations to compensate for differences in tissue thickness. An injected/control ratio was calculated for cell number to control for any possible differences in staining intensity/antibody penetration between the different animals and inter-subject variability.

### Quantitative autoradiography

#### Tissue preparation for [^3^H] DTBZ autoradiography

Procedures were adapted from Frey K et al., [20]. Thawed tissue sections were pre-incubated for 5 minutes at 25°C in potassium phosphate buffer with EDTA at pH 8.0 and then incubated for 30 minutes at 25°C in potassium phosphate buffer containing 7 nM (+)-[^3^H] DTBZ (American Radiolabled Chemicals, Inc., St. Louis, MO; specific activity 20 Ci/mmol) in the presence (nonspecific binding) or absence (total binding) of 10 µM unlabeled “cold” tetrabenazine. Sections were rinsed 3 times in fresh potassium phosphate buffer and once briefly in distilled water at 4°C to remove buffer salts. The slides were air-dried overnight and analyzed on a Beta Imager (BioSpace, Paris, France) for 24 hours in the presence of [^3^H] standards (American Radiolabled Chemicals, Inc., St. Louis, MO).

#### Tissue preparation for [^3^H] WIN 35,428 autoradiography

Tissue sections were pre-incubated for 20 min at 4°C in phosphate buffer with EDTA at pH 7.4. Sections were then incubated for 1 hr at 4°C in buffer containing 5 nM [^3^H] WIN 35,428 (New England Nuclear/Perkin Elmer, Boston, MA; specific activity 85.9 Ci/mmol) in the presence (nonspecific binding) or absence (total binding) of 10 µM nomifensine (Sigma Chemical, St. Louis, MO). Sections were rinsed twice in fresh phosphate buffered saline at 4°C and air-dried overnight and analyzed on a Beta Imager for 24 hours in the presence of [^3^H] standards [21].

#### Quantitative analysis

Quantitative analysis was performed with the program β-Vision Plus (BioSpace, Paris, France) to demarcate the anatomical regions of interest and measure radiolabeled uptake in tissue in cpm/mm^2^. A reference curve was obtained with each Bioimager analysis using [^3^H] standards containing known amounts of radioactivity. This permitted conversion of the tissue sections measures from cpm/mm^2^ to nCi/mg tissue. The specific activity of each radioligand was used to convert these measures to femtomoles per milligram tissue. Specific binding was determined by subtracting nonspecific binding values from the total binding values, measured in adjacent sections.

For [^3^H] WIN 35,428 and [^3^H] DTBZ, saturation binding analyses were conducted over a range of radioligand concentrations between 0.25 and 15 nM. Linear regression analysis of the data was used to determine the equilibrium dissociation constant (K_d_) and the maximum number of binding sites (B _max_) using Scatchard analysis for each radioligand. Since K_d_ for each radioligand did not change between control and severely affected tissues at the highest dose of MPTP, we subsequently used a single concentration (5 nM for [^3^H] WIN 35,428 and 7 nM for [^3^H] DTBZ) that was about 3 times the K_d_ of [^3^H] WIN 35,428 (1.5 nM) and of [^3^H] DTBZ (2.7 nM). We then could calculate Bmax from these data having demonstrated that K_d_ remained constant.

### Quantification of DA

Striatal level of DA was measured using high performance liquid chromatography with electrochemical detection (HPLC-EC) [22]. The HPLC system consisted of an ESA Coulochem III electrochemical detector (ESA, Dionex Company, Chelmsford, MA) with ESA model 584 HPLC pump. The conditioning cell was ESA model 5021A set at −450 mV, and the microdialysis cell was model 5014B set at 400 mV. The optimal electrode potentials for each compound were determined by current-voltage curves. Sample preparation consisted of homogenizing the previously frozen striatal tissue in glass homogenizers on ice in 450 ul of cold 0.1 M perchloric acid containing 0.4 mM sodium metabisulfite, 0.1 mM ethylenediaminetetraacetic acid (EDTA) and 50 µl of 3,4 dihydroxybenzylamine as an internal standard. The samples were centrifuged for 10 minutes at 1500× g at 4°C. The supernatant was filtered using a 0.22 micron filter spun at 15,000× g for 5 minutes and 20 µl of the filtrate was injected onto the HPLC for analysis. The internal standard permitted quantification of DA. The values were expressed as ng/gm of brain tissue.

### Statistical analysis

Results were analyzed using SPSS for Windows, version 18.0.2 (IBM, Chicago. IL). All *in vitro* measures of VMAT2 Bmax, DAT Bmax, SN cell counts and striatal DA were expressed as a ratio between the values obtained on the MPTP lesioned side and that of the contralateral side (injected/control side) to minimize the effect of biological variability among animals. We compared the residual binding density of VMAT2 and DAT with Wilcoxon signed ranks test since these data were not normally distributed demonstrated by Shapiro-Wilks test of normality [23]. The relationships of VMAT2 Bmax, DAT Bmax, and striatal DA among each other and to nigral cell counts were determined with Spearman correlations due to a non-normal distribution of data with some clustering near zero in severely affected animals. In a separate analysis, we excluded the clustered data to permit analysis with Pearson correlations in the remaining 8 monkeys. A two-tailed *P* value of less than 0.05 was considered significant.

## Results

All 14 monkeys completed the study protocol successfully and were euthanized at 56 to 60 days after MPTP infusion. Animals were always able to care for themselves despite not receiving any anti-parkinsonian drugs for the duration of the study. Animals manifested a wide range of severity of parkinsonism from none in controls to severe unilateral parkinsonism with the highest doses of MPTP.

### Striatal DAT and VMAT2 and nigral DA neuron distribution in control and MPTP-treated brain

**Figure 2:**
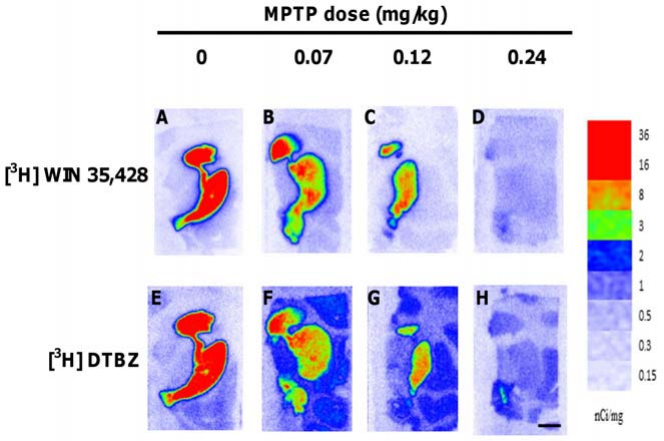
Representative autoradiograms of [^3^H] WIN 35,428 and [^3^H] DTBZ. [^3^H] WIN 35,428 binding to DAT (top row) (A–D), [^3^H] DTBZ binding to VMAT2 (bottom row) (E–H) in coronal sections from the injected side of control (A and E) and MPTP-treated monkeys (B–D and F–H). Images represent total binding, according to the pseudo-color bar on the right side of each image. Scale bar: 0.5 cm.

We performed DAT and VMAT2 autoradiography in brain tissue from all animals. The binding patterns of [^3^H] WIN 35,428 reflecting DAT and [^3^H] DTBZ reflecting VMAT2 control animals appeared consistent with previous reports in monkey and human brain. [24]–[27] (Figure 2A and E). The dorsal portions of both the caudate and the putamen exhibited the highest binding densities, whereas ventral striatal regions, including the nucleus accumbens, displayed lower binding densities. The general striatal binding patterns of the two radioligands in MPTP treated monkeys also appeared similar to that of controls (Figure 2 B–D and F–H) although the specific binding densities were variably reduced. Nonspecific binding was minimal for both radioligands (<10% of total binding).

We utilized stereology to quantify TH-ir neuron count in SNpc in 13 monkeys. One animal was excluded from the nigral cell count measures since the nigral tissue was damaged during processing. The distribution of TH-ir SNpc neurons did not appear to change as a function of MPTP dose, although the number of TH-ir SNpc neurons decreased.

### Relationship between VMAT2 and DAT binding density and SNpc TH neurons

**Figure 3:**
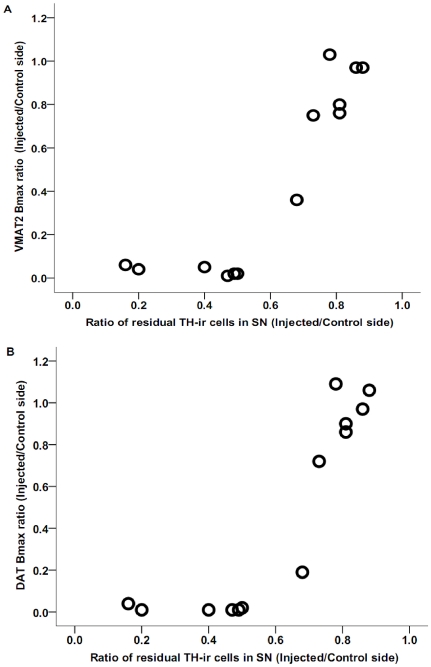
Relationship between VMAT2 (A) or DAT (B) Bmax and residual SNpc TH-ir neurons. The value for each monkey was expressed as the ratio of the injected side to the control side in 13 monkeys.

Examination of the distribution of the data in Figure 3 reveals a flooring effect for the VMAT2 and DAT binding density in animals with more than 50% nigral cell loss. Excluding those animals with more than 50% nigral cell loss revealed a significant correlation between the cell count ratio and Bmax ratio (injected/control side) for VMAT2 and DAT in the remaining animals (*r* = 0.93, *n* = 8, *p* = 0.001and *r* = 0.91, *n* = 8, *p* = 0.002 respectively).

### Correlation between VMAT2 and DAT binding density and striatal DA

**Figure 4:**
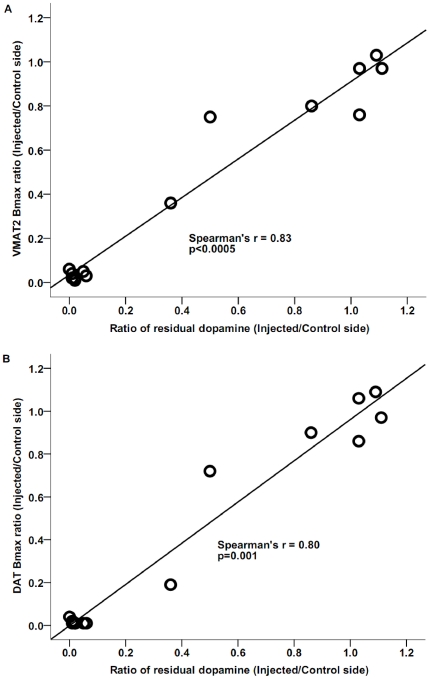
Relationship between VMAT2 (A) or DAT (B) Bmax and residual striatal dopamine content. The values for each monkey were expressed as the ratio of the injected side to the contralateral side, and the line is the linear fit of the data.

Six animals with greater than 50% neuronal cell loss had near zero striatal dopamine measures similar to their striatal VMAT2 and DAT specific binding. The data from the other 8 animals had a broad distribution permitting a Pearson correlation analysis despite clustering of the other values near zero. There was a strong significant correlation between residual striatal DA and VMAT2 as well as with DAT specific binding with or without the 6 clustered data points. (Spearman's correlation with all data: *r_s_* = 0.83, *n* = 14, *p*<0.0005 and *r_s_* = 0.80, *n* = 14, *p* = 0.001 respectively; Pearson's correlation without the clustered points: *r* = 0.94, *n* = 8, *p* = 0.001 and *r* = 0.95, *n* = 8, *p*<0.0005 respectively) (Figure 4).

### Similar loss of the DAT and VMAT2 binding after MPTP treatment

**Figure 5:**
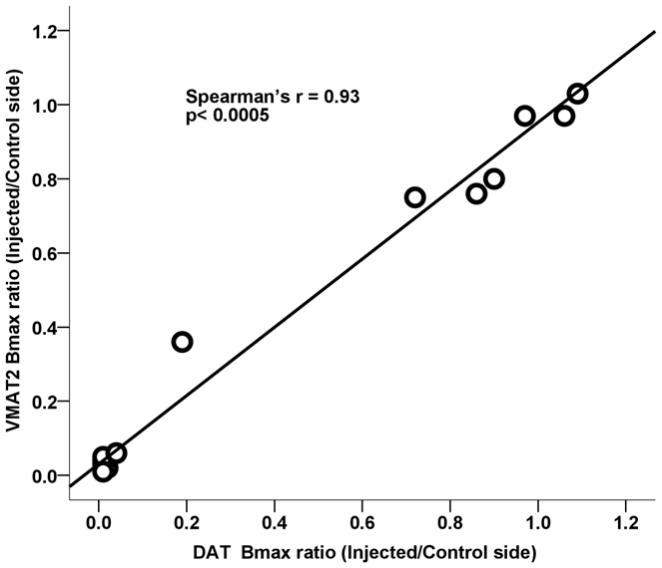
Relationship between striatal VMAT2 and DAT Bmax in 14 monkeys. The value for each monkey was expressed as the ratio of the injected side to the control side, and the line is the linear fit of the data. The r value reflects the Spearman's correlation coefficient value obtained in striatum.

The loss of striatal DAT and VMAT2 binding sites was similar following a wide range of MPTP doses and was not statistically different from each other (Wilcoxon signed ranks test *z*(14) = −0.089, *p* = 0.93). There was a strong, significant correlation between Bmax of VMAT2 and DAT with or without the data clustered near zero (Spearman's correlation with all data: *r_s_* = 0.93, *n* = 14, *p*<0.0005; Pearson's correlation without the clustered points: *r* = 0.98, *n* = 8, *p*<0.0005) (Figure 5).

## Discussion

The present study demonstrates that DAT and VMAT2 striatal binding sites do not have differential regulation across a wide range of nigral cell loss. Our data differ from the findings of postmortem studies in PD patients [3], which report greater loss of striatal DAT than VMAT2 radioligand binding. *In vivo* studies with molecular imaging methods such as positron emission tomography (PET) can permit direct comparisons of DAT and VMAT2 binding. Numerous PET studies have compared some combination of fluorine-18-L-dihydroxyphenylalanine (FD), reflecting aromatic amino-acid decarboxylase activity and DA storage, a DAT and a VMAT2 radioligand in the same PD patients [5], [7], [28], [29] with inconsistent findings. Interpretation of data from human studies can be complex. Differences in subject selection criteria, the presence of other disorders, wide disparities in length of drug exposure, limited spatial resolution of PET, effects of postmortem delay on measures of dopamine [30], [31] and variable specificity of radioligands can all influence the findings. It has, therefore, been difficult to discern the exact nature of regulation of different dopaminergic markers in humans.

Nonhuman primate models, nevertheless, have the connectivity and structural complexity homologous to the human brain [32] and offer significant advantages. Indeed the animal model allowed us to make measurements of presynaptic dopaminergic markers and neuronal counts in SNpc at different severities of nigrostriatal injury in a more controlled manner than would be possible in humans. In addition, the unilateral infusion strategy permitted use of an internal control thereby minimizing the effects of inter-subject variability. The major limitation of the MPTP model is that MPTP pathophysiology is not identical to idiopathic PD [32]. Although there may be some similarities in mechanism of cell death [33] (such as mitochondrial energy defects [34], free radical damage [35]) or possibly excitotoxicity [36]; MPTP may preferentially damage terminal fields since its toxic metabolite 1-methyl-4-phenyl-2,3-dihydropyridium ion (MPP+) is taken up by DA neurons via DAT [37]. However, preferential terminal loss in striatum compared to DA cell body loss in SNpc has been described in humans with PD [38] as well as with MPTP treated animals [39], [40]. Thus, conservatively one could view MPTP as primarily a model of dopaminergic cell loss rather than as a model of the pathophysiology of human PD. Nevertheless, this model is well-suited for the purpose of this study, to investigate whether differential regulation of DAT and VMAT2 occurs across a wide range of degrees of dopaminergic cell loss. All animals were subjected to the same psychologically enrichment tasks as required for care of nonhuman primates [41]. It is unknown whether any of the tasks that we employed could have induced neurogenesis, as some tasks may or may not have this effect [42], [43]. However, in our animal model system such effect would not alter the relationship between VMAT2 and DAT. Furthermore such an effect, if it occurred, was not sufficient to interfere with the dose-dependent effect of MPTP that we found.

We found a strong correlation between VMAT2 and DAT binding densities and postmortem measures of striatal DA levels but only with the number of SNpc dopaminergic neurons as long as the degree of SNpc neuronal loss was limited. The correlation with striatal DA agrees with other studies (for VMAT2 [6] and for DAT [44]). The loss of TH-ir SNpc DA neurons correlated well with reduction in striatal DAT and VMAT2 binding density as long as at least 50% of nigral cells were preserved; greater loss appears to be associated with a flooring effect for DAT or VMAT2. This greater degree of terminal field loss exceeding nigral cell body loss suggests that destruction of terminals fields may precede cell body loss, but we do not have time-dependent measures to prove this. We found no evidence consistent with axonal sprouting containing additional DAT or VMAT2 sites that would potentially reduce the preferential loss of terminal field markers compared to SNpc DA cell bodies [45], [46]; the compensatory responses that others reported for the acute loss of the TH-ir fibers and terminals include increased synthesis of TH, increased DA release and turnover, and decreased DA uptake.

Interestingly, one previous study also investigated the relationship between striatal FD uptake and stereologic counts of nigral dopaminergic neurons in animals with graded MPTP lesions [47]. That study limited analysis to those animals that had no more than a 35% loss of nigral neurons and found a significant correlation between FD uptake and striatal dopamine content, but no significant correlation to nigral cell counts. The limited nigrostriatal injury and poor distribution of data included in that study may contribute to the discrepancy with our study that included a wider range of injury.

In summary, our data demonstrate no differential regulation of striatal DAT compared to VMAT2 specific binding sites across a wide range of severity of nigrostriatal neuronal loss in MPTP-treated monkeys. This raises important questions about interpretation of molecular imaging studies in humans that compare different radiotracers of nigrostriatal neurons in disorders such as idiopathic PD or other neurodegenerative conditions that affect nigrostriatal pathways. Do differential results with different radiotracers reflect differences in noise properties or biases of the imaging techniques [48]–[50]; are there differential effects from previous drug exposures or differences in timing of changes in these different molecular targets? Our data strongly suggest that differential regulation of these sites is not a likely cause of disparate findings with the different radiotracers. We only examined changes in DAT and VMAT2 at 2 months after MPTP-induced injury; however, we previously demonstrated that the clinical effects of the lesions in this animal model are stable for as long as 1½ years [12] making it less likely that our findings would change with longer observation times. Short-term studies, on the other hand, could determine whether there is lack of differential regulation earlier in the course of nigrostriatal injury as well as whether terminal fields are damaged prior to nigral cell bodies. This will be important for extending the applicability of these different radiotracers as potential biomarkers of nigrostriatal neurons.
